# Transfer of FRozen Encapsulated multi-donor Stool filtrate for active ulcerative Colitis (FRESCO): study protocol for a prospective, multicenter, double-blind, randomized, controlled trial

**DOI:** 10.1186/s13063-022-06095-1

**Published:** 2022-02-22

**Authors:** Andreas Stallmach, Philip Grunert, Johannes Stallhofer, Bettina Löffler, Michael Baier, Jürgen Rödel, Michael Kiehntopf, Sophie Neugebauer, Dietmar H. Pieper, Howard Junca, Andrea Tannapfel, Ute Merkel, Ulrike Schumacher, Maria Breternitz-Gruhne, Tabitha Heller, Anja Schauer, Michael Hartmann, Arndt Steube

**Affiliations:** 1 Department of Internal Medicine IV, Jena University Hospital, Friedrich Schiller University of Jena, Am Klinikum 1, 07747 Jena, Germany; 2 Institute of Medical Microbiology, Jena University Hospital, Friedrich Schiller University, Jena, Germany; 3 Institute for Clinical Chemistry and Laboratory Diagnostics and Integrated Biobank Jena, Jena University Hospital, Jena, Germany; 4 Microbial Interactions and Processes Research Group, Helmholtz Centre for Infection, Braunschweig, Germany; 5 Institute for Pathology, Ruhr University, Bochum, Germany; 6 Hospital Pharmacy, Jena University Hospital, Jena, Germany; 7 Center for Clinical Studies Jena (ZKS), Jena University Hospital, Jena, Germany

**Keywords:** Fecal microbiota transfer, Fecal microbiota transplantation, Ulcerative colitis, Randomized controlled trial

## Abstract

**Background:**

Ulcerative colitis (UC) is a chronic inflammatory bowel disease with significant morbidity and mortality. Although the precise cause remains unknown, disturbances in the intestinal microbial community have been linked to its pathogenesis. Randomized controlled trials in UC and relapsing *Clostridioides difficile* infection (CDI) have established fecal microbiota (FM) transfer (FMT) as an effective therapy. In this context, preliminary results indicated that the transfer of sterile fecal microbiota filtrates (<0.2 μm; FMF, FMFT) of donor stool also drives gastrointestinal microbiota changes and eliminates symptoms in CDI patients. However, along with the success of FMT, regulatory agencies issued safety alerts following reports of serious adverse events due to transmission of enteric pathogens through FMT. To reduce this risk, we established an extensive test protocol for our donors and quarantine regulations for the produced capsules, but alternative concepts are desirable.

**Methods:**

Our project is a randomized, controlled, longitudinal, prospective, three-arm, multicenter, double-blind study to determine the safety and efficacy of repeated long-term, multi-donor FM or FMF transfers compared to placebo using oral, frozen capsules in 174 randomized patients with mild to moderate active UC. The primary outcome will be clinical remission at week 12.

**Discussion:**

This proposal aims to examine (a) the efficacy of encapsulated transfer of FM and FMF as a therapy for mild to moderate UC, (b) the short- and long-term safety of FMT and FMFT in patients with UC, and (c) the microbial and immunologic changes that occur after FMT and FMFT to help understand how and why it affects inflammatory bowel disease.

**Trial registration:**

ClinicalTrials.govNCT03843385. DRKS (Deutsches Register für Klinische Studien) DRKS00020471

## Strengths of this study

- This multicenter study is the first randomized three-armed placebo-controlled trial assessing the clinical efficacy of sterile fecal microbiota filtrate transfer (<0.2 μm; FMFT) for treating active ulcerative colitis compared with classical fecal microbiota transfer (FMT).
- Videotaped colonoscopy with biopsies will be performed at study entry and 12 weeks after randomization to assess endoscopic and histological inflammatory activity. Central reading of video recordings will be performed.
- Patient’s reported quality of life indices will be assessed.
- Use of oral, frozen, encapsulated fecal microbiota or fecal microbiota filtrate for long-term treatment may increase patients’ acceptance.

## Background

Ulcerative colitis (UC) is a progressive chronic inflammatory disease affecting the colon with significant morbidity and mortality. Its incidence has been increasing, and currently, the highest incidence is reported in Northern Europe (24.3 per 100.000) [1]. In Germany, about 170,000–210,000 patients are affected by UC [2]. A recently published meta-analysis demonstrated an increased standardized mortality ratio of 1.19 (95% confidence interval, 1.06–1.35) for UC patients compared to the general population [3]. Disturbances of the intestinal microbiota have been linked to the pathogenesis of UC, and in active UC, the microbiota profile differs in composition and is less diverse than that in healthy subjects [4–6]. The efficacy of FMT for recurrent CDI has spurred its application in UC. To date, a total of 26 studies (8 randomized studies, 18 cohort studies) with 587 patients with UC have been published in the last few years. Two of the 8 RCTs had negative results [7, 8], while six randomized, placebo-controlled trials had positive results [9–14]. Since the diversity of the donor microbiota and the number of taxa transferred was linked to the success of the therapy in post hoc analyses [15], a so-called multi-donor approach was chosen in recent studies in which the microbiota of different donors are pooled [10, 11]. New developments in FMT include the application of efficient frozen material [16] and the application of stool via capsules [17, 18]. Very recently, a pilot study suggests that daily encapsulated FMT may extend the durability of FMT-induced changes in the gut bacterial community structure and clinical response to FMT in UC [12]. However, larger trials should be performed to explore the benefits of FMT and to determine its long-term impacts on clinical parameters for UC.

An important issue of FMT is safety particularly in immunocompromised patients. Transfer of undefined living microorganisms entails uncontrollable risks for infections and other complications. Therefore, Schreiber and colleagues investigated whether a sterile filtrate of fecal microbiota (FMF) (containing bacterial debris, proteins, antimicrobial compounds, metabolic products, and nucleic acids) rather than intact bacteria could be used as an alternative strategy [19]. They demonstrated in a small cohort of five patients with recurrent CDI that FMF transfer (FMFT) was sufficient to restore normal bowel habits, to change the gastrointestinal microbiota, and to eliminate symptoms. As FMFT alone sufficiently altered microbial and viral community composition, it indicates that bacterial components, metabolites, or bacteriophages could mediate the effects of the classical transfer of the complete fecal microbiota. Therefore, FMFT may represent a highly attractive approach in immunocompromised patients with UC.

## Methods/design

### Study design

This is a randomized, controlled, longitudinal, prospective, three-arm, multicenter, double-blind study to determine the safety and efficacy of repeated long-term, multi-donor FMT or FMFT compared to placebo using oral, frozen capsules in 174 randomized patients with mild to moderate active UC. The primary outcome will be clinical remission at week 12.

### Intervention

In Germany, the Federal Institute for Drugs and Medical Devices (German: BfArM = Bundesinstitut für Arzneimittel und Medizinprodukte) and the leading Ethics committee of the Friedrich Schiller University Jena, together with the Ethics committees of the participating study sites, are responsible for protocol approval. If changes are made in the course of the trial, the affected documents are adapted accordingly. If any documents requiring approval are affected (e.g., the protocol), respective amendments are prepared and submitted to the federal authority and the ethics committees for approval. Only after receipt of the approval or positive vote, the procedures of the trial are adapted accordingly.

All protocol and GCP violations are documented together with the corresponding measures and reported to the sponsor’s authorized representative, who assesses the deviations and can evaluate them with additional measures if necessary. Major protocol deviations and GCP violations are immediately forwarded to the sponsor’s authorized representative and escalated to the sponsor if necessary. If any changes in the trial affect the contents of the study registries (clinicaltrials.gov and the German Registry for Clinical Studies; German: DRKS = Deutsches Register für Klinische Studien), these will be adapted accordingly.

The sponsor or sponsor’s legal representative is supported by the center for clinical studies (CCS) in the coordination of the trial. Here, the areas of data management, monitoring, biometrics, SAE, and project management are applied. Meetings of the above-mentioned areas will take place regularly during the course of the trial. The different areas of the CCS are available daily for the study centers, e.g., check the inclusion of patients in the study and do on-site monitoring and central monitoring strategies on a quarterly basis. There is also a quarterly risk review of the study. Additionally, a central study coordinator is available for all study sites and coordinates e.g. the request for the IMP from the manufacturer and the delivery of the IMP to the sites/patients. In the local study sites, the organization of the trial and the recruitment of patients under GCP conditions is carried out by local study coordinators and physicians. These are in close contact with the central study coordinator and the CCS.

During the conduct of the clinical trial, a weekly exchange takes place with all trial staff of the coordinating team involved (e.g., legal representative, project management, monitoring, data management, biometrics, pharmacovigilance, central study coordination, manufacturer, etc.). Significant study-relevant information, results, and measures are recorded. The possibility of escalation to the sponsor in case of critical and serious faults in the course of the trial is described in a CCS internal SOP.

An external Data Monitoring and Safety Board (DMSB), composed of 5 international experts in inflammatory bowel disease and one patient representative, will review the progress of the study. Interim reviews of safety data will be performed. Recommendations whether the nature, frequency, and severity of adverse effects associated with study treatment warrant the early termination of the study in the best interests of the participants, the study should continue as planned, or the study should continue with modifications will be provided by the DSMB. The initial meeting of the DMSB took place 6 months after the approval of the federal authority and before the beginning of the trial. It continues to be conducted at the time the study is approved (yearly), and in addition, at least every 6 months thereafter during the trial. The DMSB is independent from the sponsor and members declare that they have no competing interests.

The preparation of frozen capsules for FM-, FMF-, and placebo-treatment will be carried out in the pharmacy of the University Hospital Jena under GMP conditions. The consistent preparation of capsules is guaranteed by using concerted standard operating procedures and identical conditions according to one shared GMP certificate. This trial will be carried out in Germany at approximately 20 study sites (list of study sites and further information can be obtained from the principal investigator Prof. Dr. A. Stallmach). All study sites have the required experience in clinical studies in the field of chronic inflammatory bowel diseases and treat patients regularly. Patients who meet the inclusion criteria (see below) have to consent to the FRESCO study and will be randomized 1:1:1 to receive intensive dosing of multi-donor FMFT or classical FMT as therapeutic strategies or saline as a placebo comparator. Before randomization, a screening endoscopy (with collection of a biopsy) will be performed and videotaped for central reading. Stool samples will be taken for determination of fecal calprotectin level and microbiome and virome profiling. In addition, patients will be assessed for Inflammatory Bowel Disease Questionnaire (IBDQ) [20] and concomitant medication (CM), and a physical examination (PE) will be performed. Adverse events (AE) will be assessed using the Common Terminology Criteria for Adverse Events.

**Fig. 1:**
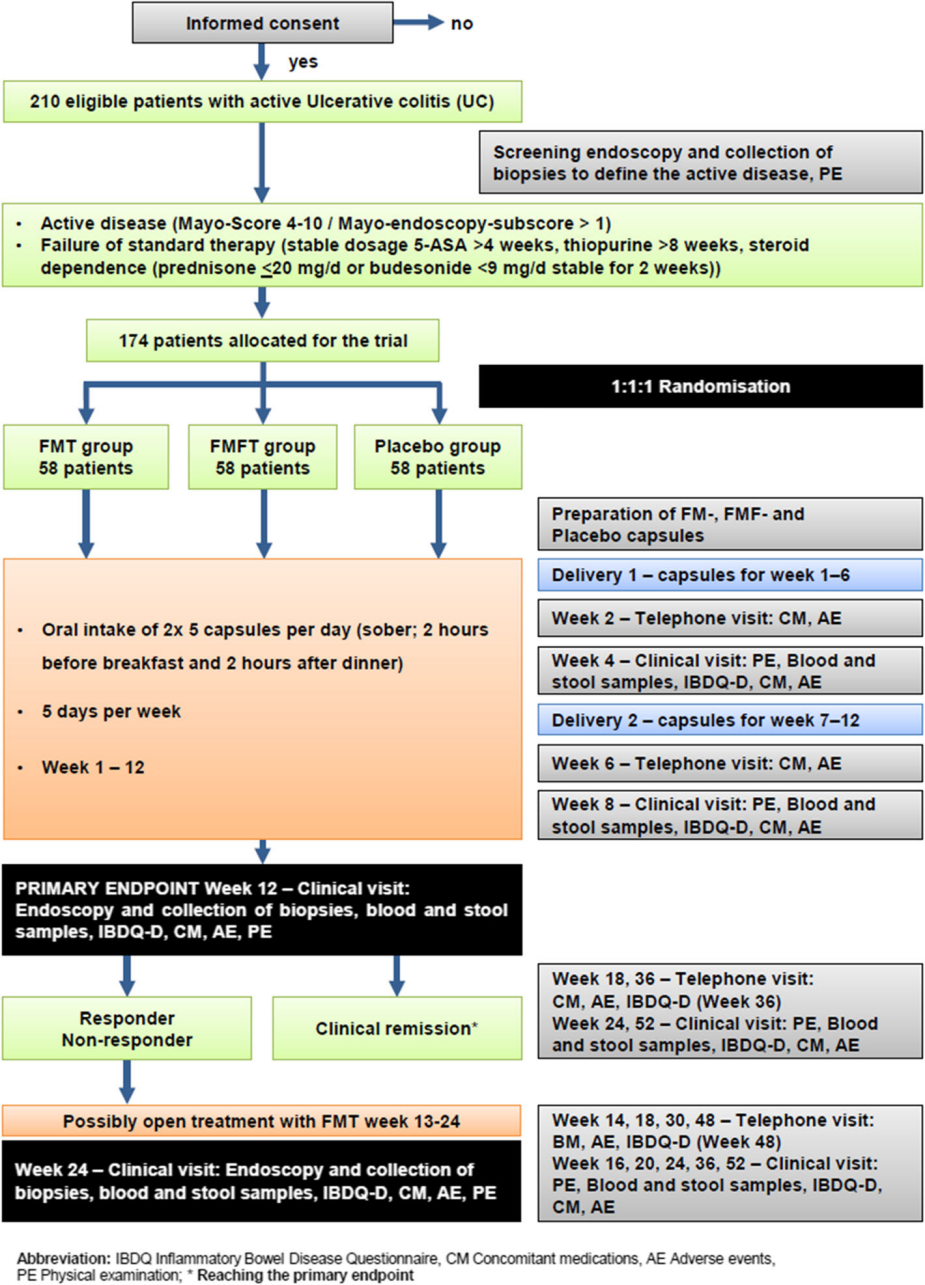
Intervention scheme FRESCO study

The prepared frozen capsules will be handed out and transferred to the patients in cooling boxes at study week 0 (start point) and week 6. The capsules need to be refrigerated (−15°C ± 5°C) by the patient in order to maintain the pharmacologic activity of FM and FMF. Patients will take 2 × 5 frozen capsules (morning/evening) on 5 consecutive days per week (5 days on and 2 days off; week 1–week 12) with cold liquid (e.g., water) (Fig. 1).

### Patient population

#### Inclusion criteria

- 18–75 years old
- Written informed consent
- Prior endoscopic confirmation of active UC of at least 6 months *and* with a minimum disease extent of 15 cm from the anal verge
- Having active disease, defined with a Mayo Score between 4 and 10 and Mayo endoscopic subscore >1 at study entry
- May be receiving the following drugs (subjects on these therapies must be willing to remain on stable doses for the noted times)
- Oral 5-ASA compounds provided the dose prescribed has been stable for at least 4 weeks prior to randomization; the dose must be stable for the first 12 weeks after randomization
- Azathioprine, 6-MP, or MTX provided the dose prescribed has been stable for 8 weeks prior to randomization; the dose must be stable for the first 12 weeks after randomization
- Oral corticosteroid therapy (prednisone prescribed at a stable dose ≤ 20 mg/day or budesonide prescribed at a stable dose of ≤ 9 mg/day) provided the dose prescribed has been stable for 2 weeks prior to randomization
- Topical therapy (foams, enema) with mesalazine or budesonide: the dose prescribed has been stable for 2 weeks prior to randomization
- Complete vaccination against SARS-CoV-2 as recommended by the STIKO
- Ability to understand and willingness to sign informed consent document in patients whom the investigator believes can and will comply with the requirements of the protocol

#### Exclusion criteria

- Crohn’s disease or indeterminate colitis or proctitis alone
- Acute abdomen or other clinical emergencies requiring emergent management (e.g., bowel obstruction, perforation and/or abscess, previous bowel surgery)
- Concurrent gastrointestinal infections
- Other causes of diarrhea
- Congenital or acquired immunodeficiency, severe comorbidities (e.g., diabetes mellitus, cancer, systemic lupus, decompensated cirrhosis, recent malignancy in the last 3 years)
- Negative EBV/CMV serology
- Pregnancy
- Patients who are unable or unwilling to undergo colonoscopy
- Previous treatment with anti-TNF, integrin, or IL12/IL23 antibodies within the last 8 weeks
- Previous treatment with calcineurin or JAK inhibitors within the last 4 weeks
- Systemic antibiotic use within the last 8 weeks
- Participation in a clinical trial within the last 3 months
- Prior history of FMT or FMFT
- Probiotic use within 14 days of the start date
- Not ensuring frozen storage (−15°C ± 5°C) of the capsules
- Addictive or other medical conditions or circumstances that do not allow the subject to appreciate the nature, significance, scope, and possible consequences of the clinical trial
- Signs of non-compliance of the participant

### Recruitment

The numerous inquiries about FMT from patients in different treatment centers express the high level of interest shown on this complementary form of treatment. The trial has a multicenter character, and all participating study sites carried out an individual sample size estimate in advance. Taking into account the inclusion and exclusion criteria, approximately 600 patients are suitable. Furthermore, study calls for example via different organizations and networks will increase the number of eligible patients. This strategy will ensure an adequate number of participants to achieve the planned sample size. The recruitment period is 2 years from the start of the trial. The study is conducted for patients who are able to give informed consent, after a physician of the study group provided oral and written information using the forms provided. The patient gets information about the screening and all further required measurements, the nature, significance, objective, duration, procedure, benefits, all risks and other aspects of the clinical trial, and the use of the investigational medicinal product (IMP). Only after the patient has given informed consent, screening for participation in the trial and the measurements required for this purpose can start.

### Randomization

The assignment of the patients to one of the 3 treatment arms is randomized. After initiation of each study site, patients will be consecutively screened and eligible patients who are willing to participate will be included in the FRESCO trial. The patients are stratified according to the study site and previous therapy with biologics (yes/no). Each study site receives a list of consecutive randomization numbers for each of the two strata relating to the treatment. These will be allocated in the order of arrival of the patient and documented in the electronic database (eCRF). The manufacturer keeps an unblinded list per study site and previous therapy with biologics (yes/no). Upon request by a study site, the manufacturer will prepare the trial medication for the patient according to the treatment specified in the randomization list. The patient is blinded with respect to the medication. To address “concealment of allocation,” the 1:1:1 randomization will be done centrally and each patient who is randomized and who received one of the compared treatments is part of the full analysis set and analyzed according to the ITT (intention to treat) principle. To achieve balanced distributions for pretreatment factors, we apply stratified (factor 1: no steroids/steroids/thiopurines/steroids and thiopurines; factor 2: “participating centre”) block randomization. Emergency unblinding takes place by calling the 24-h housekeeping service at the Clinic for Internal Medicine IV, Jena University Hospital.

### Compliance strategies and monitoring

Intervention protocol compliance strategies and compliance monitoring procedures are in place, e.g., patients keep a diary. Besides describing their health and stool condition and reporting the presence of blood in the stool, patients have to record the daily/weekly capsule intake. Additionally, they return all remaining capsules they have not taken in course of the trial to the study sites as well as all empty tubes at the end of the trial. There are no laboratory tests planned, to investigate compliance.

### Outcomes

#### Primary outcome

The primary outcome will be clinical remission at week 12 post first transfer of FMT or FMFT, defined by Mayo score ≤ 2 and without any subscore >1; additionally, patients who are unavailable at follow-up week 12 will be included as non-responders (i.e., counted no remission). The Mayo Score/Disease Activity Index (DAI) is a validated scoring system for the assessment of ulcerative colitis activity including stool frequency, rectal bleeding, endoscopic findings, and physician’s global assessment [21].

#### Secondary outcomes/safety

Secondary outcomes will be steroid-free clinical remission, clinical response (defined by a decrease in Mayo score by 3 points, with a decrease in bleeding subscore by 1 as an important patient-related outcome parameter, or absolute Mayo score of 0–1), quality of life (assessed with IBDQ), and safety (assessed by adverse events).

##### Mucosal inflammation

To assess mucosal inflammation, fecal calprotectin and histologic remission as further secondary endpoints will be measured at week 12 and compared to week 0. Histologic remission will be assessed using the Geboes histologic scores [22] composed of 6 different grades and the Nancy histological index [23] for evaluation of disease severity in UC.

##### Patient-reported outcome measures/health-related quality of life (HRQOL)

Subjects will complete the IBDQ at the time points specified in the schedule of events (weeks 0, 4, 8, 12, 24, 36, and 52). The IBDQ is a valid and reliable instrument used to assess the quality of life in adult patients with IBD [20]. It includes 32 questions on 4 domains of HRQOL: Bowel Systems (10 items), Emotional Function (12 items), Social Function (5 items), and Systemic Function (5 items). Subjects are asked to recall symptoms and quality of life from the last 2 weeks and rate each item on a 7-point Likert scale (higher scores equate to a higher quality of life). A total IBDQ score is calculated by summing the scores from each domain; the total IBDQ score ranges from 32 to 224.

##### Microbiome/virome profiling

The microbiome composition will be characterized based on high-throughput sequencing of amplicons of the V1V2 regions of the 16S rRNA gene. This region has a high-resolution power, and in various genera, it allows a differentiation down to the species level [24]. Analysis will be performed by sequencing on the Illumina MiSeq platform with at least 30,000 sequences per sample. Raw data will be processed with an established bioinformatical pipeline and processed data analyzed for its microbial diversity and changes in the abundance of phylotypes, genera, and families [25]. Furthermore, the donor FM will be analyzed for bacterial composition and FM and FMF will be subject to virome analysis. In brief, virus-like particles (VLPs) will be purified as previously described [26]. Libraries will be generated from VLP DNA using the TruSeq® Nano DNA Library Preparation Kit and sequenced on the Illumina MiSeq platform at a sequencing depth of 2,000,000 paired end sequences. Assemblies will be done using de novo assembly tools such as Metavelvet [27]. Identification and taxonomic classification of viruses will be performed by recently developed applications such as Metavir [28]. VLPs will also be analyzed from recipient stool and stool after FM or FMF transfer to identify if bacteriophages are transferred and established in the recipient.

##### Adverse events

To assess adverse events, the Common Terminology Criteria for Adverse Events (CTCAE) will be used.

### Stopping rules

#### Procedures for discontinuation or withdrawal of a patient

The investigator may discontinue a subject’s study participation at any time during the study when the subject meets the study termination criteria described below. In addition, a subject may discontinue his or her participation without giving a reason at any time during the study.

#### Disease worsening criteria

Interruption of treatment with the investigational medicinal product is required in the case of a worsening of the underlying disease defined as follows:

- Increase in the partial MAYO score (all components excluding the endoscopic subscore) by ≥ 3 points on two consecutive visits compared to the screening visit: Visits include planned study visits as well as unplanned visits to the primary care physician, another physician, or the study site), e.g., a study visit followed by a visit to the primary care physician or 2 consecutive unplanned visits during the treatment phase
- Worsening of the underlying disease that, in the opinion of the investigator’s treating physician, leads to the use of an unauthorized concomitant medication
- Occurrence of acute abdomen or other clinical emergency (toxic megacolon, fulminant gastrointestinal hemorrhage, ileus, perforation, etc.)
- Occurrence of an acute gastrointestinal infection (e.g., CDI, CMV infection)

#### Interruption of treatment for other medical reasons

Interruption of treatment with the IMP is required in the case of:

- Occurrence of an opportunistic infection or other infections requiring hospitalization
- Indication for systemic antibiotic therapy (independent of the indication)
- Occurrence of an acute illness which, in the opinion of the attending physician of the study group, significantly impairs the patient’s overall well-being, e.g., fever > 38.5°C persisting for more than 72 h, symptomatic anemia (e.g., shortness of breath, palpitations, weakness, fatigue)
- If necessary, for planned or emergency surgical procedures (not e.g. for mini-surgeries on the skin or teeth under local anesthesia or without anesthesia)

#### Criteria for premature termination for the whole trial

The sponsor representative is entitled to interrupt or prematurely terminate the entire clinical trial, e.g., if:

- The recruitment rate is inadequate
- Serious, unresolvable problems arise with the quality of the data collected
- Unacceptable risks have arisen (decision after a new risk-benefit assessment has been made)
- New scientific findings during the duration of the clinical trial do not allow it to be continued
- There could be a risk to patient safety

#### Provisions for post-trial care

The further treatment of participants after the regular end of their participation, after withdrawal of their consent, or in case of discontinuation of the treatment or the clinical trial is carried out according to the national standards defined in guidelines. Medications for relapse prophylaxis, which were already taken during the clinical trial as concomitant medication in permitted dosage and not newly initiated, will be continued in unchanged dosage according to the standards. Further treatment will be continued at the study site or with the previously treating specialist.

### Sample size

Sample size calculations were performed for the primary outcome remission rates 12 weeks post first FMT/FMFT/placebo. The sample size calculation is based on a 2×3 table *χ*^2^ test as implemented in nQuery Advisor 7.0 (*χ*^2^ test of equal proportions in G groups (equal *n*’s)) even though a more complicated model will be used for the confirmatory analysis. Based on this, we estimate the remission rates 12 weeks post first FMT/FMFT to be *π*_FMT_ ≈30% or π_FMFT_ ≈30% while *π*_Placebo_≈5% are expected under placebo. To detect such a clinically relevant benefit at an overall power of 1 – *β* = 0.8 for a global significance level of *α* = 0.05 (two-sided), a sample size of *n* = 3 × 40 = 120 is required for the confirmatory analysis. To account for a potential dropout rate of 30% overall and/or lower power of the more advanced analysis model, the total sample size is *n* = 3 × 58 = 174 patients.

Table 1 shows the impact of slightly altered planning assumptions regarding the true rates on the global power. Moreover, the table also depicts power estimates for the joint test of FMT/FMFT against placebo and the two-group tests of FMT vs. placebo, FMFT vs. placebo (two group continuity corrected *χ*^2^ test of equal proportions; unequal and equal *n*). As similar rates are expected for FMT and FMFT, no power estimates are provided for this comparison.

**Table 1:** Statistical power analysis

True rates [%]			Sample size per group (including dropouts)^a^	Power for the respective test setting [%]		
*π*_FMT_	*π*_FMFT_	*π*_Placebo_		Global test^b^	FMT and FMFT vs. placebo	FMT or FMFT vs. placebo
30	30	5	58	93	98	92
30	40	10	58	92	95	69 rsp 95
40	40	10	58	96	99	95

^a^Resulting in a total sample size would be 3 × 58 = 174, 3 × 93 = 279, and 3 × 50 = 150 patients to be randomized

^b^The increased power (i.e., a power larger than 80%) results from the inclusion of dropouts in the analysis

### Statistical analysis

The main analysis will be conducted in the full analysis set according to the intention-to-treat principle. Remission rates will be compared with a two-step hierarchical testing procedure. In a first step, a generalized linear mixed model (GLMM) with fixed effects treatment and steroid/thiopurine stratum and random effect site will be applied to test the null hypothesis H_0_ that no difference in remission rates will be observed between the groups against the alternative in which there will be a difference between the groups. In case of rejection of H_0_, the following tests will be performed in a hierarchical order:

1. The FMFT and FMT group combined versus placebo
2. FMT versus placebo
3. FMFT versus placebo
4. FMFT versus FMT

by means of Wald statistic on a two-sided significance level of 0.05. The significance level is met by this hierarchical testing approach.

Further sensitivity analyses in a per-protocol set, explorative subgroup analyses according to stratification levels, and analyses of secondary parameters with a respective GLMM approach are described in detail in the study protocol.

In principle, all data will be analyzed at least by descriptive statistics, i.e., number of available and missing data, mean, standard deviation, minimum, quartiles, and maximum for metric data and frequency analysis for categories.

## Discussion

The primary purpose of this study is to assess the effect of an Investigational Medicinal Product (IMP) intervention in patients with mild to moderate active ulcerative colitis. A stable engraftment of the transferred multi-donor microbiome or a stable modification of the patient microbiome is expected from long-term application over 12 weeks. The application of frozen encapsulated fecal microbiota (FM) or sterile fecal microbiota filtrate (FMF) allows a non-invasive at-home treatment which increases patient acceptance and compliance.

Numerous logistical challenges have to be resolved before starting the clinical study. In this context, patient safety is most important. Transmission of enteropathogens by FMT is a key safety issue (and feared complication). In 2019, two immune-compromised patients contracted ESBL *E. coli* infection after FMT from a common stool donor [29]. The Federal Institute for Drugs and Medical Device (BfArM) issued a safety alert in June 2019 regarding the use of FMT and the risk of serious adverse reactions due to transmission of multidrug-resistant organisms and screening for these pathogens was made standard practice after these instances worldwide [30, 31]. While exclusion testing for multi-resistant pathogens is established, the reliable exclusion of a clinically inapparent SARS-CoV-2 infection in donors is a challenge. Through the combination of repeated testing of potential stool donors (repeated nasopharyngeal swabs and stool examinations) with quarantining of the processed stool donation in a stool bank achieved the greatest possible safety. This concept is reinforced by accepting only COVID-19-vaccinated stool donors and participating patients. The frozen oral FM- or FMF-capsules are released from quarantine after the second tests of stool donors, 8 weeks after the first screening was passed.

The definition of threshold values for the microbiome diversity of the donors and a high similarity between the FM of the donor and the processed encapsulated FM act as an inclusion criterion for donors and as a quality standard for the production of FM-capsules. The definition of quality standards for the frozen FMF was resolved by setting a limit for the quotient for the concentration of bile acids in the dispenser stool versus that in the filtrate. However, the time-consuming setup of a mass spectral analysis of bile acids was necessary.

The delivery of the frozen IMP turned out to be a further logistical problem. Concerns regarding freeze–thaw cycles incurred by transport, the use of dry ice during the transport process, and home freezer conditions were addressed. According to the German Medicinal Products Act, IMPs may not be delivered from the manufacturer to the patient directly. Thus, a two-stage time-fixed delivery transport from the manufacturer to the patient with an in-between quality control in the test center by the study investigator was planned via a certified logistics company.

A major motivation for carrying out this clinical trial is the great interest shown by patients in this treatment concept. From numerous discussions with patients and representatives of self-help organizations, it becomes clear that they expect a more causal, but — above all — low-side-effect treatment approach in the future. Therefore, the patient’s perspective was already taken into account when planning the study. The current trial could therefore assign a concept of targeted microbiota modification as a future long-term treatment strategy of UC avoiding immunosuppressive therapy.

## Trial status

Protocol version number and date: version 03, 23.09.2021

The FRESCO trial is expected to start recruitment from May 2022 and recruitment is expected to finish in May 2024.

## Data Availability

Those involved in the FRESCO study have access to the final trial dataset and, upon request, the dataset can be made available to the participating test centers. The results will be uploaded to the EudraCT database and can also be published by the DRKS and clinical trials (register for studies). There are no restrictions regarding a publication of the data. Data protection for the patients is guaranteed.

## Abbreviations

AE: Adverse events
BfArM: Federal Institute for Drugs and Medical Device
CCS: Center for clinical studies
CDI: *Clostridioides difficile* infection
CM: Concomitant medication
CMV: Cytomegalovirus
COVID-19: Coronavirus disease 2019
CTCAE: Common Terminology Criteria for Adverse Events
DMSB: Data Monitoring and Safety Board
DRKS: German Registry for Clinical Studies
EBV: Epstein–Barr virus
FM(T): Fecal microbiota/microbiome (transfer)
FMF(T): Fecal microbiota/microbiome filtrate (transfer)
GCP: Good Clinical Practice
GMP: Good Manufacturing Practice
GLMM: Generalized linear mixed model
IBDQ: Inflammatory Bowel Disease Questionnaire
IMP: Investigational Medicinal Product
ITT: Intention to treat
PE: Physical examination
SARS-CoV-2: Severe acute respiratory syndrome coronavirus 2
SOP: Standard operating procedure
UC: Ulcerative colitis
VLPs: Virus-like particles
